# Supplementary material for: Splenic Elemental Composition of Breast Cancer-Suffering Rats Supplemented with Pomegranate Seed Oil and Bitter Melon Extract
Source: Molecules. 2024 Apr 24;29(9):1942. doi: 10.3390/molecules29091942 (PMC11085740; doi:10.3390/molecules29091942)
Supplement: Supplementary file 1 [file molecules-29-01942-s001.zip › molecules-2940026-supplementary.pdf]

Table S1. Values of limit of detection (LOD) and limit of quantitation (LOQ) of detected minerals.

	LOD [mg/kg]	LOQ [mg/kg]	LOD [ug/L]	LOQ [ug/L]
		macroelements		
K	2.216	2.244	8.36	8.49
Mg	0.513	0.525	0.92	0.93
Na	11.353	11.537	8.95	9.16
Ca	1.812	1.829	8.72	8.84
		microelements		
Fe	0.827	0.858	0.76	0.85
Zn	0.276	0.278	1.90	1.92
Cu	0.243	0.246	1.67	1.68
Mn	0.008	0.009	0.00	0.00
Se	0.014	0.023	0.83	0.85
Co	0.001	0.001	0.08	0.08
Cr	0.067	0.068	0.52	0.52
Ni	0.027	0.028	0.37	0.37
Al	11.285	11.368	12.20	12.20
Sr	0.020	0.021	0.73	0.74
Pb	0.045	0.046	0.62	0.63
Cd	0.001	0.001	0.10	0.10
B	0.311	0.328	5.27	5.36
Tl	0.003	0.004	0.39	0.39

Table S2. Chemical characteristic of dietary supplements.

**Fatty acid [µg/g]**	**BME**	**PSO**
C6:0	nd	7.63
C8:0	nd	10.55
C10:0	nd	4.05
C12:0	0.12	11.63
C14:0	0.41	50.9
C15:0	0.16	26.2
cis7 C15:1	nd	5.85
C16:0	2.42	6555
cis7 C16:1	0.34	26.3
*cis*9 C16:1	nd	8.31
C17:0	0.15	155
*cis6*C17:1	nd	nd
*cis*9C17:1	nd	8.77
C18:0	6.58	6483
*trans*11C18:1	nd	24.7
*cis*9 C18:1	0.58	12170
*cis*11 C18:1	nd	833
*cis*14 C18:1	nd	7.73
*cis*9*cis*12 C18:2 (LA)	nd	16748
*cis*6*cis*9cis12 C18:3 (GLA)	nd	23.5
cis8cis11cis14 C18:3 n-4	nd	nd
*cis*9*cis*12*cis*15 C18:3 (ALA)	0.42	1466

C20:0	0.12	1003
*cis*11C20:1	nd	nd
C21:0	nd	60
C22:0	nd	250
C24:0	nd	100
Σ CLnA:	0.00	111000
*cis*9*trans*11*cis*13 C18:3 (PA)	nd	45414
*cis*9*trans*11*trans*13 C18:3 (αESA)	nd	16280
Conjugated fatty acids:		
ΣCFA:	2.00	723481
ΣCD:	0.23	nd
tt isomers	0.23	nd
ct/*tc* isomers	nd	nd
cc isomers	nd	nd
ΣCT:	1.77	723481
ttt isomers	1.58	546525
ttc isomers	0.11	68660
cct isomers	0.07	108296
	**BME 1% aqueous extract**	**BME 10% methanolic extract**
Compound [mg/100g dw.]:		
Chlorogenic acid	6.33	956
Caffeic acid	nd	26.0
Sinapic acid	nd	58.5
Isochlorogenic acid	nd	87.6
Rutin	tr	tr
Protocatechuic acid	nd	tr
Luteolin	nd	tr
Kaempferol	nd	tr
Epigallocatechin	nd	tr
Quercetin	nd	tr
Quercitrin	nd	tr
Total polyphenol content [g/l]	640	1489
DPPH [µmol Trolox/l]	290	851
FRAP [µmol Fe2+/ l]	654	154
	**PSO**	
IV [mgI$_2$/100g oil]	120	
PV [mEq O/kg oil]	5.74	
AV [mg KOH/g oil]	6.94	

cis9cis12 C18:2 – linoleic acid (LA); cis6cis9cis12 C18:3 – γ-linolenic acid (GLA); cis9cis12cis15 C18:3 – α-linolenic acid (ALA); cis9trans11cis13 C18:3 – unicic acid (PA); cis9trans11trans13 C18:3 – α-eleostearic acid (αESA); CFA – conjugated fatty acids, CD – conjugated dienes, CT – conjugated trienes, cc – cis,cis isomers, ct/tc – cis,trans/ trans,cis isomers, tt – trans,trans isomers, ttt – trans,trans,trans isomers, ttc – trans,trans,cis isomers, cct – cis,cis,trans isomers, AV - acidic value; DPPH - 1,1-diphenyl-2-picrylhydrazyl; FRAP - ferric reducing antioxidant power; IV - iodine value; PV - peroxide value; 0.00 – amount was below the quantification limit (<LOQ); nd – not detected; tr – trace amount.
